# Physiological resistance alters behavioral response of *Tetranychus urticae* to acaricides

**DOI:** 10.1038/s41598-019-55708-4

**Published:** 2019-12-17

**Authors:** Adekunle W. Adesanya, Michael J. Beauchamp, Mark D. Lavine, Laura C. Lavine, Fang Zhu, Doug B. Walsh

**Affiliations:** 1 Irrigated Agriculture Research and Extension Center, Washington State University, Prosser, WA 99350 USA; 2 Department of Entomology, Washington State University, Pullman, WA 99164 USA; 3 Department of Entomology, Pennsylvania State University, State College, PA 16803 USA

**Keywords:** Behavioural ecology, Evolution

## Abstract

Multiple acaricide resistance in *Tetranychus urticae* continues to threaten crop production globally, justifying the need to adequately study resistance for sustainable pest management. Most studies on acaricide resistance have focused on the acute contact toxicity of acaricides with little or no information on the behavioral responses elicited after acaricide exposure. Furthermore, the impact of physiological resistance on these behavioral responses remains unknown in most pest species, including *T. urticae*. We tested the effect of acaricide resistance on contact toxicity, irritancy and repellency of mitochondrial electron transport inhibitor of complex I (MET-I) and mite growth inhibitor (MGI) acaricides on multiple *T. urticae* strains. We also tested whether acaricides with similar physiological target site/mode of action also elicit similar behavioral effects on *T. urticae* strains. MET-I acaricides (fenazaquin, fenpyroximate, and pyrabiden) and MGIs (clofentezine, hexythiazox and etoxazole) elicited a dose-dependent irritant and repellent effect on *T. urticae*. Selection of strains for physiological resistance to these acaricides affected the behavioral response of *T. urticae*, especially in MET-I resistant strains, that showed reduced irritancy and repellency to MET-I acaricides. Behavioral response also affected the oviposition of *T. urticae*, where strains generally showed preferential oviposition away from the acaricides. The outcome of this study highlights negative consequences of acaricide resistance that can potentially affect *T. urticae* management.

## Introduction

In addition to a pesticide’s direct lethality, its sublethal effects can also affect its efficacy^1^. For example, pesticides can act as repellents or irritants, causing pests to leave crop plants before causing mortality. This could result in undesirable consequences if it causes pest dispersal within and across crops leading to increased damage^2,3^. Hence the need to characterize the lethal and sublethal effects of currently available pesticides used for the management of pests of medical, urban, and agricultural importance. Presently, most prescribed procedures for testing the efficacy or pest resistance to pesticides as recommended by the Insecticide Resistance Action Committee^4^ entail laboratory assays that force the target pest to have direct contact with the candidate pesticide: dose-response assays with 100% spray coverage or no choice feeding studies. These methods are inherently artificial compared to most field settings, as it is almost impossible to achieve complete pesticide coverage due to intrinsic factors including crop architecture, timing of application, weather and micro climate effects, etc.^5^ Pesticide-induced behavioral changes have been reported for some pests such as mosquitoes^2^, horn flies^6^, lepidopteran pests such as diamond back moths^7,8^, beet armyworm^9^, and urban pests including the German cockroach^10^. An important class of agricultural pests, phytophagous mites have received very limited attention with respect to the sublethal effects of acaricide exposure^11^, especially for the non-neurotoxic acaricides. Data on the sublethal effects of registered and formulated acaricides will improve their effective use in mite management programs.

Adaptation to pesticides by numerous pests has been well documented for many economically important arthropod pests^12–18^. The mechanisms of pesticide resistance have been described to fall into four main categories: target site insensitivity, metabolic detoxification, reduced penetration and behavioral resistance. The first three mechanisms are collectively termed as physiological resistance^7^ because they generally involves heritable changes in pest physiological status that reduce the toxicity of a pesticide’s active ingredients after coming in physical contact with the target pest species. A lot of empirical evidence has shown that various mechanisms of physiological resistance contribute to pesticide resistance in most studied arthropod species^15,16,18–22^. However, there is paucity of studies that have investigated the contribution of behavioral resistance in arthropod adaptation to pesticides. A landmark study of behavioral resistance in the German cockroach was able to link changes in gustatory receptors in a resistant population (glucose-aversive) to behavioral resistance^23^. Characterizing behavioral resistance in arthropods is a challenging task because it involves providing empirical evidence of heritable traits that are “*evolved behaviors to reduce an arthropod’s exposure to toxic compounds or that allow an insect to survive in what would otherwise be a toxic and fatal environment”*^24,25^. This is opposed to *behavioral avoidance* which involves a learned ability by the arthropod to avoid a life threatening situation based on previous experience. Another problem is how to empirically separate *behavioral resistance* i.e. stimulus-dependent avoidance which is the pesticide-induced avoidance of contact from *behavioral avoidance* which is a form of stimulus-independent avoidance such as typical habits or behavioral patterns of a pest that result in natural reduction or avoidance of being exposed to pesticides^26,27^. More importantly, it is not clear whether the development of physiological resistance (target site mutation/metabolic detoxification/reduced penetration) can affect a pest’s behavioral response to a pesticide.

The two-spotted spidermite, *Tetranychus urticae* Koch, is a highly polyphagous pest of global distribution with a host range of over 1100 plant species. Among the host plants of *T. urtica*e are highly valued economic crops of agricultural and horticultural importance^28–32^. The control of *T. urticae* is largely dependent on the use of multiple acaricides with different modes of action. Because of its attributes of short generational time, high fecundity, arrhenotoky, diapause, and extensive polyphagy, *T. urticae* has developed resistance to almost all the acaricides available for its control^15,33^. Physiological mechanisms of resistance are well documented in multiple acaricide-resistant *T. urticae* strains^14–16,32^. However, behavioral components of resistance of *T. urticae* to acaricides have received little attention^5,6^. Moreover, the consequence of acaricide resistance development in *T. urticae* and other species has been based on fitness related traits including population growth^9,34,35^ and cross resistance to other acaricides^28^, but not on the sensory perception of acaricides by *T. urticae*. It is unknown whether acaricide resistant *T. urticae* populations may have developed an acaricide-aversive behavior or become desensitized to the presence of an acaricide since they have developed physiological mechanisms to cope with its acute toxicity.

In this study, we focused on investigating the behavioral responses (irritancy and repellency) and oviposition behavior of acaricide susceptible and resistant strains of *T. urticae* to mitochondrial complex I electron transport inhibitors (fenpyroximate, pyrabiden, fenazaquin) and mite growth inhibitors (clofentezine, etoxazole, hexythiazox). We tested whether the development of physiological acaricide resistance to these acaricides influenced *T. urticae*’s behavioral response including its impact on oviposition.

## Methods

### Spidermite strains

An acaricide susceptible strain of *T. urticae* (**SUS**) was used as the reference strain for this study. This strain was collected from a hopyard in Prosser, WA in summer 2015 and through bioassay exposure determined to be relatively susceptible to acaricides^28^. A cohort of **SUS** mite strain was subjected to selection pressure from commercial formulated acaricides to develop high levels (resistance ratios >100-fold) of acaricide resistance for at least 24 months. The laboratory acaricide selection generated near-isogenic acaricide resistant *T. urticae* strains: FUJI_RS, NEXT_RS, MAG_RS, APOLLO_RS, SAVEY_RS and ZEAL_RS that are highly resistant to Fujimite SC (fenpyroximate), Nexter SC (pyrabiden), Magister SC (fenazaquin), Apollo SC (clofentezine), Savey DF (hexythiazox) and Zeal WP (etoxazole) respectively (Table 1). All the *T. urticae* strains described above were maintained on live lima bean plants *Phaseolus vulgaris* L. and were raised under controlled conditions (25 ± 2 °C, 70 ± 5% RH, and a photoperiod of 16:8 (Light: Dark).

**Table 1:** Active ingredient, formulation and concentration of acaricides used in this study.

Active ingredient (a.i.)	IRAC group	Trade name	% a.i. and formulation	*labelled rate ppm a.i.
Fenpyroximate	MET-I, 21A	Fujimite	5 SC	250
Fenazaquin	MET-I, 21A	Magister	18 SC	540
Pyrabiden	MET-I, 21A	Nexter	42 SC	600
Clofentezine	Mite growth inhibitor (10A)	Apollo	5 SC	626
Etoxazole	Mite growth inhibitor 10B	Zeal	72 WP	300
Hexythiazox	Mite growth inhibitor 10A	Savey	50 DF	460

*Based on the highest field recommended dose on the acaricide label.

### Acaricides evaluated

Six commercially available formulated acaricides were used in this study. We were attempting to mimic actual field conditions that *T. urticae* could encounter in mite susceptible crops. The acaricides were either mitochondrial complex I electron transport inhibitors (MET-I) i.e., Fujimite SC, Nexter SC, Magister SC, or mite growth inhibitors (MGI) i.e., Apollo SC, Savey DF and Zeal WP. Low (0.1X), moderate (0.5X), and high (1X) concentrations of each acaricide were tested on the *T. urticae* strains, with X representing the maximum labeled rate for the acaricide. Acaricide dilutions were made in double distilled water and were used within 48 h for mortality and behavioral assays (Table 1).

### Contact toxicity assays

The acute toxicity of the MET-I acaricides in this study was tested on adult females from the acaricide-susceptible ‘SUS’ strain, the MET-I resistant strains (FUJI_RS, NEXT_RS, and MAG_RS) and one MGI-resistant strains (ZEAL_RS). The acute toxicity test for each MGI acaricide was also on the susceptible ‘SUS’ strain, three MGI-resistant strains (APOLLO_RS, SAVEY_RS and ZEAL_RS) and one MET-I resistant strain (NEXT_RS). Leaf disc bioassay arenas (2 cm in diameter) were created on water-soaked cotton placed in petri dishes. The cotton materials were extended to cover-up the edges of the leaf discs to prevent mites from escaping and ensure they had maximum contact with the acaricides applied by a laboratory sprayer (Potter spray tower, Burkard Manufacturing, Richmansworth, Herts, UK) with aqueous deposit of 2.0 ± 0.1 mg/cm^2^ spray fluid. The bioassay arenas were kept in an incubator (Percival scientific, Perry, IA, USA) at the conditions mentioned above. The tested doses of each acaricide were the high, moderate and low doses, as well as the control (distilled water). For the MET-I acaricides, each dose was replicated six times on a group of adult female mites (12–15). The number of dead and survivor mites in each replicate was counted under a dissecting microscope at 48 h after treatment. For the MGI acaricides, which are primarily ovicidal/larvicidal, approximately 8–10 mated adult females of each *T. urticae* strain were placed on a leaf disc and permitted to lay eggs for 24 h (15–30 eggs), after which the adult females were removed and bioassay arenas (circular leaf discs) were sprayed with varying doses (low, moderate and high) of the acaricides. Each dose of MGI acaricide was also replicated six times. Mortality of mites was scored after 5–7 days, after the eggs sprayed with distilled water (controls) had hatched.

### Behavioral assays

**Figure 1:**
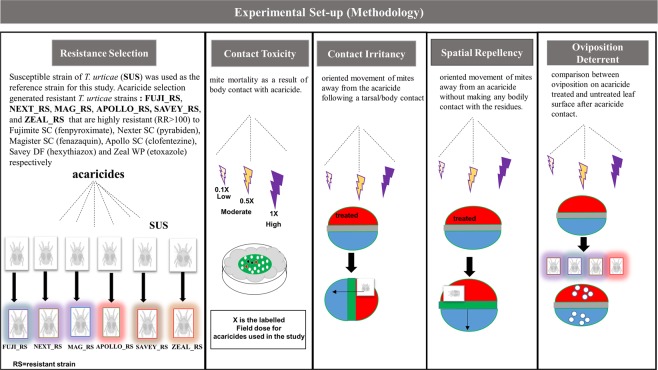
Schematic outline of the experimental set-up.

Three major behavioral effects of acaricide exposure that can impact their biological efficacy were evaluated. These factors include contact irritancy, spatial repellency and oviposition^8,10^. These bioassays were designed to mimic the high-throughput screen system for mosquitoes^35^. Our working definitions of contact irritancy and spatial repellency were adapted from Roberts *et al*.^36^. Contact irritancy is the oriented movement of mites away from the acaricide following a tarsal/body contact, and spatial repellency is the oriented movement of mites away from an acaricide chemical without making any bodily contact with the residues of the acaricide. In order to evaluate the contact irritancy of MET-I acaricides, 2 cm-diameter leaf discs were made from fresh lima bean leaves with the midrib forming the diameter, similar to Beers *et al.*^37^. Half of the circular leaf-disc was tightly covered with a glass slide, while the other half of the leaf disc was then treated with the appropriate dose of acaricide using a laboratory sprayer (Potter spray tower, Burkard Manufacturing, Richmansworth, Herts, UK). The sprayed leaf discs were air-dried in a fume hood for 1 h on paper towels. Gravid adult female mites (for MET-I acaricides) or larval mites (for MGI acaricides) were placed in the center of the treated half of the leaf disc. Preliminary observation with distilled water treatment indicated mites from both susceptible and acaricide resistant strains were equally dispersed on a water-treated half of leaf disc within 4 h. At least 12 mites were assayed per individual leaf disc and each dose of acaricide was replicated at least 5 times. Mites that moved away from the treated half of the leaf disc to the untreated side were scored as ‘*irritated*’ while mites that remained in the treated arena were scored as ‘*non-irritated*’ Spatial repellency of the candidate acaricide at each dose was evaluated with a similar design to the contact irritancy assay. However, rather than placing the mites on the treated half of the leaf disc, larvae or adult female mites were placed on the mid-rib section of the leaf-disc (Fig. 1) and then the mites were allowed to move onto the treated or untreated halves of the leaf disc. Preliminary observations with distilled water treatments showed that mites exhibited no bias to either water-treated or untreated areas. Just as with the irritancy assays, mites’ location was quantified 4 h after placement on the leaf disc.

High fecundity is a major factor in the biological success of *T. urticae*, hence it is important to know if acaricide avoidance behavior like irritancy is accompanied with higher fecundity. This study quantified oviposition deterrence of METI and MGI acaricides by comparing the fecundity of *T. urticae* strains on acaricide treated (non-irritated mites) and untreated leaf arenas (irritated mites) at varying low, moderate and high doses. The design of the fecundity assays was similar to the irritancy assays. In the fecundity assays, 8–10 young (~3 day old) adult female mites were allowed to move between acaricide-treated and untreated leaf disc halves after being initially placed on the treated half (Fig. 1). The number of eggs laid by mites on the treated and untreated halves of the leaf discs was counted after 24 h with the aid of a dissecting light microscope. This experiment was replicated six times for each acaricide dose.

### Statistical analysis

All statistical analyses were conducted in R studio version 3.4.4^36^. For datasets investigating acute toxicity (mortality) and behavioral response (irritancy and repellency), the response variables were binary data (dead or alive, irritated or non-irritated, repelled or non-repelled). A general linear model (glm) with a binomial function was used to test for the significant effect of acaricide dose, resistance status (strains) and their interactions (dose*strain) on the acute toxicity, irritancy and repellency of mites across the tested *T. urticae* strains. Within each *T. urticae* strain, effect likelihood ratio test was used to separate the average number of dead, irritated or repelled mites at the tested doses and significance was set at α = 0.05. In order to better understand the relationship between physiological resistance and behavioral avoidance, a correlation analysis was performed on direct toxicity, repellency and irritancy within each *T. urticae* strain among these parameters using Pearson’s product-moment correlation in R to estimate the coefficient of correlation and probability of obtaining it. ANOVA was used to test for the overall significant effect of dose, resistance status (strain), irritancy and their interactions on the oviposition rate (fecundity) of *T*. *urticae* within each *T. urticae* strain and across strains tested for an acaricide. Tukey-HSD post-hoc test was used for mean separation at α = 0.05.

## Results

### Contact toxicity of MET-I acaricides

**Figure 2:**
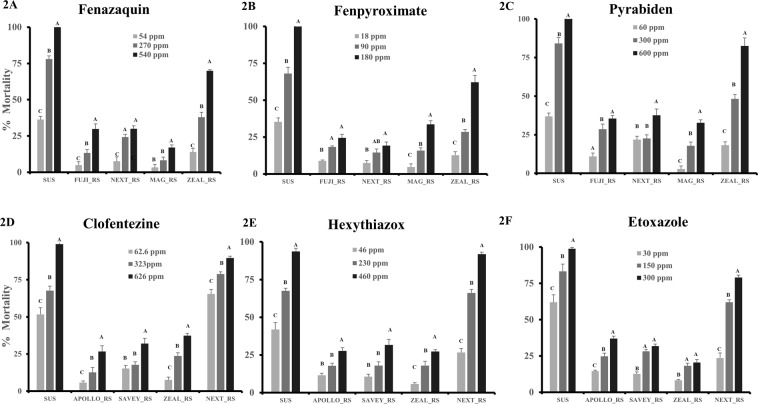
Contact toxicity (mortality) of mitochondrial electron transport inhibitors of complex I (MET-I; **A–C**) and mite growth inhibitor (MGI; **D–F**) acaricides on different *Tetranychus urticae* strains. Bars represent proportion of dead mites at each acaricide dose. Different letters above bars indicate significant differences in mite mortality (*P* < 0.05) at tested doses within each *T. urticae* strain.

The degree of toxicity (mortality) of fenazaquin to adult female mites varied significantly with dose (χ^2^ = 130, df = 2, *P* < 0.0001) and strain (χ^2^ = 281.3, df = 4, *P* < 0.0001) but not their interaction (χ^2^ = 7.2, df = 8, *P* < 0.13). As expected, the SUS (χ^2^ = 55.4, *P* < 0.0001) and ZEAL_RS (χ^2^ = 48.2, *P* < 0.0001) strains were the most sensitive to fenazaquin across the tested doses, though the mortality of the former was much higher (1.4–2.6-fold) than that of the latter, at all the tested doses (Fig. 2A). Mite mortality increased with fenazaquin doses in the SUS and ZEAL_RS strains. Mortality of the MET-I resistant strains (FUJI_RS (χ^2^ = 15.9, *P* < 0.0001), NEXT_RS(χ^2^ = 9.6, *P* = 0.002) and MAG_RS(χ^2^ = 8.2, *P* = 0.004)) was low (<30%), even at the high fenazaquin dose, evidence of similar mode of action by fenazaquin, fenpyroximate and pyrabiden.

Acute toxicity of fenpyroximate to adult female mites depended on dose (χ^2^ = 100.8, df = 2, *P* < 0.0001), strain (χ^2^ = 225.9, df = 4, *P* < 0.0001) and their interaction (χ^2^ = 15.7, df = 8, P = 0.003). SUS and ZEAL_RS strains were very sensitive to fenpyroximate treatment at all doses, although the mortality of the ZEAL_RS was at least 1.5-fold lower than that of the SUS strain at each dose. There was a significant effect of dose on mite mortality in the SUS (χ^2^ = 52.5, *P* < 0.0001), ZEAL_RS (χ^2^ = 37.6, *P* < 0.0001), MAG_RS (χ^2^ = 19.6, *P* < 0.0001), and FUJI_RS (χ^2^ = 5.3, *P* = 0.02) strains but not in the NEXT_RS (χ^2^ = 1.4, *P* = 0.24) strain (Fig. 2B).

Toxicity of pyrabiden to adult female mites of different *T. urticae* strains significantly varied by dose (χ^2^ = 125, df = 2, *P* < 0.0001), strain(χ^2^ = 245.7, df = 4, *P* < 0.0001), and also their interaction (χ^2^ = 33.7, df = 8, *P* < 0.0001). Mite mortality significantly increased with tested dose of pyrabiden in the SUS (χ^2^ = 103.4, *P* < 0.0001), ZEAL_RS (χ^2^ = 65.5, *P* < 0.0001), MAG_RS (χ^2^ = 22, *P* < 0.0001) and FUJI_RS (χ^2^ = 58.5, *P* < 0.0001) strains but not in the NEXT_RS (χ^2^ = 0.58, *P* = 0.44) strain (Fig. 2C).

### Contact toxicity of MGI-acaricides

Mortality of the tested *T. urticae* strains to clofentezine (Apollo) depended on the dose (χ^2^ = 187, *P* < 0.0001, df = 2), the resistance status of the mite (χ^2^ = 820, *P* < 0.0001, df = 4) and also interaction between dose and strain (χ^2^ = 19.9, *P* < 0.001, df = 8). There was significant effect of clofentezine dose on mite mortality in SUS (χ2 = 118, P < 0.0001), APOLLO_RS (χ2 = 10.4, P = 0.0012), SAVEY_RS (χ2 = 10.4, P = 0.0012), ZEAL_RS (χ2 = 27.8,P < 0.0001) and NEXT_RS (χ2 = 19.8,P < 0.0001). Mortality of the non-MGI resistant strain (NEXT_RS) was significantly lower to that of the susceptible strain but higher than the MGI-resistant strains (APOLLO_RS, ZEAL_RS and SAVEY_RS) across the tested doses **(**Fig. 2D).

The toxicity of hexythiazox also varied by dose (χ^2^ = 227.6, *P* < 0.0001, df = 2), strain (χ^2^ = 559, *P* < 0.0001, df = 4) and interaction between dose and strain (χ^2^ = 137.2, df = 8, P < 0.001). Mite mortality significantly varied across the tested doses of hexythiazox in all the *T. urticae* strains, SUS (χ^2^ = 81.6, *P* < 0.0001), APOLLO_RS (χ^2^ = 12.1, *P* = 0.0005), SAVEY_RS (χ^2^ = 18.4, *P* < 0.0001), ZEAL_RS (χ^2^ = 27.8, *P* < 0.0001) and NEXT_RS (χ^2^ = 125, *P* < 0.0001). Toxicity to MGI-resistant strains is relatively low at the tested doses (less than 35%). The mortality of mites caused by hexythiazox in the SUS and NEXT_RS strains was high (>50%) across the tested doses (Fig. 2E).

Mite mortality by etoxazole also varied by the dose (χ^2^ = 189.1, *P* < 0.0001, df = 2), strain (χ^2^ = 802, *P* < 0.0001, df = 4) and the interaction between dose and strain (χ^2^ = 44.4, df = 8, *P* < 0.0001). Etoxazole was approximately four times more toxic to the SUS (χ^2^ = 889, *P* < 0.0001) strain compared to the MGI-resistant strains across the tested doses. The toxicity of etoxazole to the NEXT_RS (χ^2^ = 92, *P* < 0.0001) strain also varied significantly across the doses (Fig. 2F). Mites selected for resistance show cross-resistance in contact assays to acaricides with the same mode of action.

### Contact irritancy of MET-I acaricides

**Figure 3:**
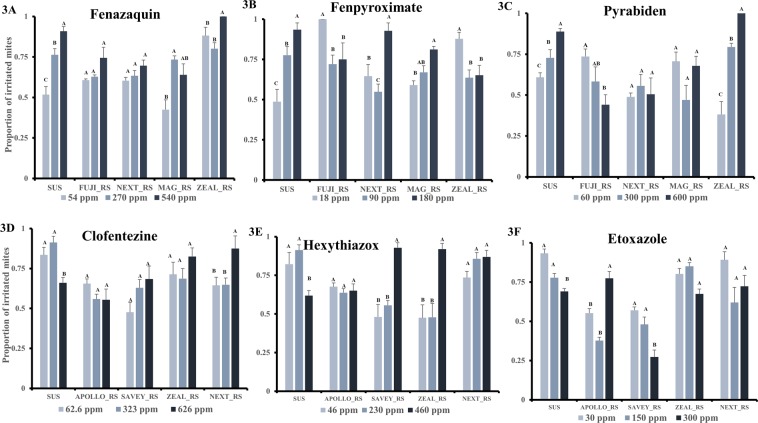
Contact irritancy of mitochondrial complex I electron transport inhibitors (MET-I; **A–C**) and mite growth inhibitors (MGI; **D–F**) on different *Tetranychus urticae* strains. Bars represent proportion of irritated mites at each acaricide dose. Different letters above bars indicate significant differences in mite irritancy (*P* < 0.05) at tested doses within each *T. urticae* strain.

The proportion of mites irritated by fenazaquin significantly varied with dose (χ^2^ = 18.7, df = 2, *P* < 0.0001), and the resistance status of the *T. urticae* strains (χ^2^ = 35.7, df = 4, *P* < 0.0001) but the interaction between dose and strain was not significant (χ^2^ = 5.6, df = 8, P = 0.23). The proportion of irritated mites increased with fenazaquin dose in the sensitive SUS (χ^2^ = 18.3, *P* < 0.0001), and ZEAL_RS (χ^2^ = 10.54, *P* = 0.05) strains, and also in MAG_RS (χ^2^ = 4.0, *P* = 0.02), while there was no dose effect on mite irritancy in the FUJI_RS (χ^2^ = 0.99, *P* = 0.61) and NEXT_RS (χ^2^ = 0.71, *P* < 0.71) strains. The proportion of irritated mites in SUS increased 1.4-fold as the dose of fenazaquin increased. There was a 1.7-fold increase in irritancy between the low and moderate dose in the resistant MAG_RS strain (Fig. 3A).

The proportion of adult female mites irritated on contact by fenpyroximate varied significantly based on dose (χ^2^ = 17.2, df = 2, *P* = 0.0002) and the resistance level of the *T. urticae* strain (χ^2^ = 28.2, df = 4, *P* < 0.0001) and by the interaction between dose and strain (χ^2^ = 52.6, df = 8, *P* < 0.0001). In SUS (χ^2^ = 23.0, *P* < 0.0001), irritancy of fenpyroximate increased with dose tested with 48.7, 77.6 and 93.5% of the tested mites irritated at the lower (18 ppm a.i.), moderate (90 ppm a.i.) and upper (180 ppm a.i.) dose, respectively. In FUJI-RS (χ^2^ = 6.9, *P* = 0.03), there was almost complete irritation (99%) by fenpyroximate at a low dose and a 72 and 75% irritation at moderate and high doses respectively. NEXT_RS (χ^2^ = 29.2, *P* < 0.0001) and MAG_RS (χ^2^ = 4.0, *P* = 0.041) strains, which have cross resistance to fenpyroximate, exhibited similar irritancy responses to fenpyroximate, with highest irritancy of 92 and 81% observed at the upper dose respectively. In ZEAL_RS (χ^2^ = 5.7, *P* = 0.045), more mites were irritated (87.8%) at the lower dose of fenpyroximate than the middle dose (63.5%) or higher dose (65.1%) (Fig. 3B).

Irritancy of pyrabiden to adult females of different strains of *T. urticae* depend on dose (χ^2^ = 4.5, P = 0.034, df = 2), strain (χ^2^ = 24.6, *P* < 0.0001, df = 4) and their interaction (χ^2^ = 45.7, *P* < 0.0001, df = 8). Increasing the dose of pyrabiden increased the proportion of mites irritated in the SUS (χ^2^ = 9.2, *P* = 0.01) and ZEAL_RS (χ^2^ = 35, P < 0.0001) strains and decreased in the FUJI_RS (χ^2^ = 6.9, *P* = 0.01) strain. There was no dose effect in NEXT_RS (χ^2^ = 2.2, *P* = 0.33) and MAG_RS (χ^2^ = 4.5, P = 0.1) strains (Fig. 3C).

### Contact irritancy of MGI acaricides

In general, the proportion of mites irritated by clofentezine varied significantly not by the dose (χ^2^ = 1.1, df = 2, *P* = 0.3), but by the mite strain (χ^2^ = 19.6, df = 4, *P* = 0.0006) and interaction between dose and strain (χ^2^ = 13.6, df = 8, *P* = 0.008). (Fig. 3D). Irritancy of mites by clofentezine varied significantly by dose within non-MGI resistant *T. urticae* strains. High doses of clofentezine reduced the proportion of mite irritated in the SUS (χ^2^ = 4.2, *P* = 0.04) strain by 1.4-fold while in NEXT_RS (χ^2^ = 5.9, P = 0.01) increased by 1.3-fold. The proportion of mites irritated in the APOLLO_RS (χ^2^ = 80.9, *P* = 0.3), SAVEY_RS (χ2 = 2.5, *P* = 0.11) and ZEAL_RS (χ^2^ = 2.3, P = 0.13) strains was not affected by the doses of clofentezine tested (Fig. 3E).

Across the tested *T. urticae* strains, the irritancy of hexythiazox varied significantly by dose (χ^2^ = 14.9, *P* = 0.0001, df = 2), strain (χ^2^ = 19.6, df = 4, *P* = 0.0005) and by their interaction (**χ**^2^ = 39, df = 8, *P* < 0.0001). There was no dose effect on the proportion mites irritated in the APOLLO_RS and NEXT_RS strains (Fig. 3E).

The irritancy of etoxazole (Zeal) among the tested *T. urticae* strains depended on the strain (χ^2^ = 55.7, P < 0.0001, df = 4), and the interaction between strain and dose (χ^2^ = 22.5, df = 8, *P* = 0.0002). There was no single main effect of dose across tested *T. urticae* strains (χ^2^ = 1.8, df = 2, *P* = 0.18). High dose of etoxazole irritated more mites in APOLLO_RS (2.1-fold) (χ^2^ = 8.9, *P* = 0.003), while the reverse was the case in SAVEY_RS (1.9-fold) (χ^2^ = 7.6, *P* = 0.01) and SUS (1.9-fold) (χ ^2^ = 2.3, *P* = 0.13). There was no dose effect on the irritancy of etoxazole in the ZEAL_RS (χ^2^ = 2.3, *P* = 0.13) and NEXT_RS (χ^2^ = 1.9, *P* = 0.17) strains (Fig. 3F).

### Spatial repellency of MET-I acaricides

**Figure 4:**
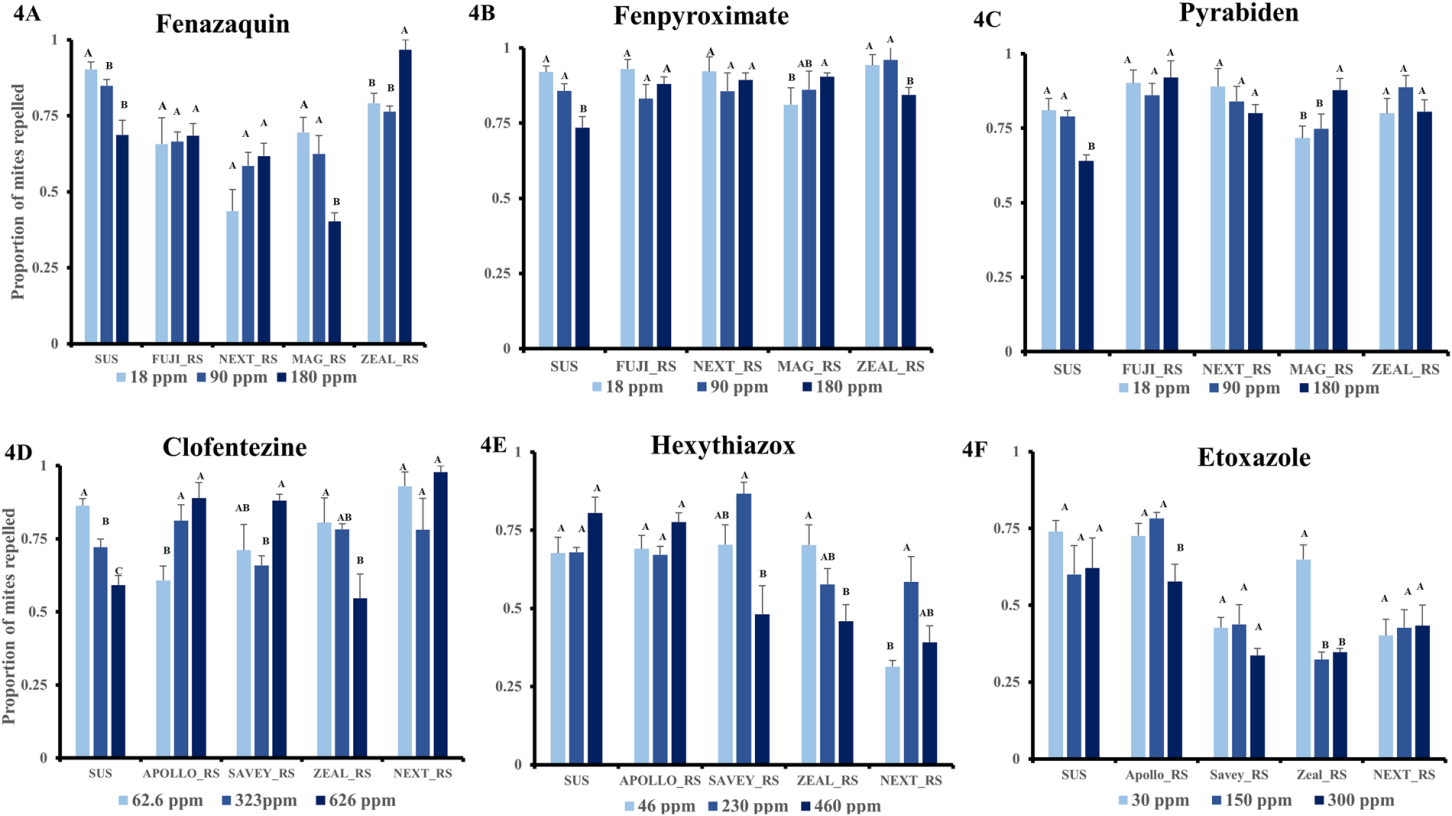
Spatial repellency of mitochondrial complex I electron transport inhibitors (MET-I; **A–C**) and mite growth inhibitors (MGI; **D–F**) on different *Tetranychus urticae* strains. Bars represent proportion of repelled mites at each acaricide dose. Different letters above bars indicate significant differences in mite repellency (*P* < 0.05) at tested doses within each *T. urticae* strain.

Generally, the proportion of mites repelled by fenazaquin depended not on the dose (χ^2^ = 0.35, P = 0.55) but on the resistance status of the mites (χ^2^ = 41.7, *P* < 0.0001) and its interaction with dose (χ^2^ = 20.2, *P* = 0.0004). There was no significant difference in the level of mite repellency by the fenazaquin doses in FUJI_RS (χ^2^ = 0.1, *P* = 0.9) and NEXT_RS (χ^2^ = 2.8, *P* = 0.3). The repellant effect of fenazaquin was significantly reduced at the high dose in the SUS (1.3-fold) (χ^2^ = 17.9, *P* < 0.0001) and MAG_RS (1.6-fold) (χ^2^ = 7.54, *P* = 0.02) strains while the opposite effect was observed in the ZEAL_RS (1.2-fold) (χ^2^ = 9.3, *P* = 0.01) strain (Fig. 4A). This suggests that *T. urticae’s* ability to perceive fenazaquin may not be directly linked with its toxicity.

In all the *T. urticae* strains, the proportion of mites repelled by fenpyroximate did not vary by dose (χ^2^ = 1.4, *P* = 0.23) or mite strain (χ^2^ = 12.8, *P* = 0.012), but there was significant interaction between dose and mite strain (χ^2^ = 8.2, *P* = 0.08). The proportion of mites repelled by fenpyroximate increased with dose in the MAG_RS (χ^2^ = 3.9, *P* = 0.04) strain and decreased in the SUS (χ^2^ = 17.9, *P* = 0.006) and ZEAL_RS (χ^2^ = 9.3, *P* = 0.07) strains. There was no significant effect of dose on mites repelled in NEXT_RS (χ^2^ = 0.4, *P* = 0.5) and FUJI-RS (χ^2^ = 0.58, *P* = 0.5) (Fig. 4B).

Pyrabiden repelled more than 50% of mites in the different *T. urticae* strains at all tested doses. Generally, the level of mite repellency by pyrabiden across different strains did not depend on dose (χ^2^ = 0.84, *P* = 0.36), but was significantly affected by strain (χ^2^ = 12.8, *P* = 0.01) and interaction between dose and strain (χ^2^ = 8.2, *P* = 0.08). The proportion of mites repelled decreased (20%) at high doses of pyrabiden in the SUS (χ^2^ = 8.2, *P* = 0.05) strain but significantly increased by 20% in the MAG_RS strain (χ^2^ = 3, *P* = 0.08). Varying the dose of pyrabiden did not significantly impact its repellency on FUJI-RS (χ^2^ = 0.26, *P* = 0.61), NEXT_RS (χ^2^ = 1.2, *P* = 0.28) and ZEAL_RS (χ^2^ = 0.83, *P* = 0.4) (Fig. 4C).

### Spatial repellency of MGI acaricides

Mite repellency by clofentezine generally varied by strain (χ^2^ = 15.9, *P* = 0.003) and interaction between strain and dose (χ^2^ = 33.4, *P* < 0.0001) but not dose alone (χ^2^ = 0.03, *P* = 0.86). In the SUS strain, the degree of irritancy decreased with the dose of clofentezine (χ^2^ = 10.5, *P* = 0.001), while it increased in the APOLLO_RS (χ^2^ = 10, *P* = 0.001) strain. There was no significant effect of dose on the repellency of clofentezine in the SAVEY_RS (χ^2^ = 4.8, *P* = 0.03), ZEAL_RS (χ^2^ = 7.2, P = 0.07) and NEXT_RS strains (χ^2^ = 0.1, *P* = 0.3) (Fig. 4D).

Across the tested *T. urticae* strains, the repellency of hexythiazox depended on *T. urticae* strain (χ^2^ = 22.1, *P* = 0.0002) and its interaction (χ^2^ = 33.4, *P* < 0.0001) with dose but not dose alone (χ^2^ = 2.7, *P* = 0.1). There was no dose effect on the proportion of mites repelled in the SUS (χ^2^ = 0.8, *P* = 0.4), APOLLO_RS (χ^2^ = 0.9, *P* = 0.3) and NEXT_RS (χ^2^ = 0.08, *P* = 0.7) strains, while few mites were repelled at the high dose of hexythiazox in the SAVEY_RS (χ2 = 5.8, *P* = 0.02) and ZEAL_RS (χ^2^ = 5.2, *P* = 0.03) strains (Fig. 4E).

Etoxazole’s repellency to the tested *T. urticae* strains was significantly affected by the dose (χ^2^ = 7.5, *P* = 0.006), the *T. urticae* strain (χ^2^ = 38.1, *P* < 0.0001) and their interaction (χ^2^ = 9.2, *P* < 0.06). Varying the dose of etoxazole did not affect its repellency in the SUS (χ^2^ = 0.6, P = 0.4), SAVEY_RS (χ^2^ = 0.7, *P* = 0.4) and NEXT_RS (χ^2^ = 0.19, *P* = 0.7) strains, while significantly fewer mites were repelled by at the high dose of etoxazole in the APOLLO_RS (χ^2^ = 7.2, *P* = 0.007) and ZEAL_RS (χ^2^ = 8.1, *P* = 0.004) strains (Fig. 4F).

In order to better understand the relationship between direct toxicity, repellency and irritancy, a correlation analysis was performed within each *T. urticae* strain for each acaricide using these parameters. The toxicity and irritancy of fenazaquin were positively correlated, though in varying degree, except in the MAG_RS strain. There was significant correlation between fenazaquin toxicity and repellency, though in different directions: positive in the NEXT_RS, and ZEAL_RS strains, and negative in SUS, and MAG_RS strains. Fenazaquin’s irritancy and repellency was significantly correlated in three *T. urticae* strains, positively in ZEAL_RS and negatively in SUS and MAG_RS strains (Table 2).

**Table 2:** Correlation between contact toxicity, irritancy and repellency of mitochondrial electron transport inhibitors (MET-I) on *T. urticae* strains.

Acaricide	*T. urticae* strain	Toxicity vs Irritancy			Toxicity vs Repellency			Irritancy Vs Repellency			
		r (CI)	t*	*P*	r (CI)	*t*	*P*	r (CI)	*t*		*P*
Fenazaquin	SUS	**0.89 (0.73–0.96)**	**7.9**	**<0.0001**	**−0.76 (−0.9** − **(−0.44))**	**−4.6**	**0.0002**	**−0.64 (−0.85** − **(−0.25))**	**−3.3**	**0.004**	
	FUJI_RS	**0.59 (0.16–0.82)**	**2.9**	**0.01**	−0.22 (−0.6 − (−0.27))	−0.9	0.37	−0.01 (−0.4–0.39)	−0.37	0.71	
	NEXT_RS	**0.49 (0.02–0.78)**	**2.2**	**0.04**	**0.53 (0.08–0.79)**	**2.4**	**0.02**	−0.02 (−0.48–0.45)	−0.07	0.95	
	*MAG_RS*	0.22 (−0.27–0.62)	0.91	0.37	**−0.59 (−0.83–0.17)**	**−2.9**	**0.01**	**−0.51 (−0.74–0.067)**	**−2.5**	**0.01**	
	ZEAL_RS	**0.83 (0.6–0.94)**	**5.9**	**<0.0001**	**0.72 (0.36–0.89)**	**3.9**	**0.001**	**0.89 (0.71–0.95)**	**7.6**	**<0.0001**	
Fenpyroximate	SUS	**0.87 (0.69–0.95)**	**7.4**	**<0.0001**	**−0.75 (−0.9** − **(−0.43))**	**−4.5**	**0.0003**	**−0.72 (−0.89** − **(−0.37))**	**−4.1**	**0.0008**	
	*FUJI_RS*	**−0.80 (−0.92** − **(−0.54))**	**−5.4**	**<0.0001**	−0.28 (−0.66 − (−0.21))	−1.2	0.26	0.26 (−0.24–0.65)	1.1	0.3	
	NEXT_RS	0.34 (−0.14–0.70)	1.5	0.15	0.12 (−0.37–0.55)	0.48	0.64	0.24 (−0.25–0.64)	0.99	0.34	
	MAG_RS	**0.84 (0.63–0.94)**	**6.3**	**<0.0001**	**0.73 (0.41–0.89)**	**4.3**	**0.0004**	0.35 (−0.14–0.7)	1.5	0.16	
	ZEAL_RS	**−0.51 (−0.79** − **(−0.01))**	**−2.4**	**0.03**	**−0.59 (−0.73–0.08)**	**−3.8**	**0.006**	**0.61 (0.20–0.84)**	**3.1**	**0.007**	
Pyrabiden	SUS	**0.84 (0.62–0.94)**	**6.2**	**<0.0001**	**−0.63 (−0.85–0.28)**	**−3.2**	**0.005**	**−0.52 (−0.79–0.07)**	**−2.4**	**0.03**	
	FUJI_RS	**−0.56 (−0.81** − **(−0.13))**	**−2.7**	**0.01**	0.42 (−0.05–0.74)	1.9	0.07	−0.14 (0.57 − (−0.34))	−0.59	0.56	
	*NEXT_RS*	0.22 (−0.27 − (−0.62))	0.9	0.38	−0.34 (−0.7-_−0.14)	−1.5	0.17	**−0.53 (−0.8–008)**	**−2.4**	**0.02**	
	MAG_RS	−0.02 (−0.48–0.44)	−0.1	0.91	**0.58 (0.15–0.82)**	**2.8**	**0.01**	0.04 (−0.43–0.50)	0.19	0.85	
	ZEAL_RS	**0.81 (0.55–0.93)**	**5.6**	**<0.0001**	−0.43 (−0.74 − (−0.04))	−1.9	0.07	−0.23 (−0.63–0.25)	−0.99	0.34	

*Degree of freedom in all cases is 16, significant correlation in bold. CI is the confidence interval for r (correlation co-efficient).

Toxicity and irritancy of fenpyroximate were significantly correlated in the tested *T. urticae* strains except in the NEXT_RS strain, with positive correlation in the SUS and MAG_RS strains and a negative relationship in the FUJI_RS and ZEAL_RS strains. Fenpyroximate’s toxicity and repellency were negatively correlated in the SUS and ZEAL_RS strains and positively correlated in the MAG_RS strain. There was no significant correlation in the FUJI_RS strain. Mite repellency and irritancy by fenpyroximate was not correlated in the FUJI_RS, NEXT_RS and MAG_RS strains. There was positive correlation in the ZEAL_RS strain and negative correlation in the SUS strain **(**Table 2).

There was strong positive correlation between pyrabiden toxicity and irritancy in the SUS and ZEAL_RS strains and negative correlation in the FUJI_RS strain. There was no correlation in the NEXT_RS and MAG_RS strains. However, the correlation of pyrabiden’s toxicity and repellency was significantly negative in the SUS strain and positive in the MAG_RS strain. There was no significant correlation in the FUJI_RS, NEXT_RS and ZEAL_RS strains. Irritancy and repellency of pyrabiden were not significantly correlated in the FUJI_RS, MAG_RS and ZEAL_RS strains but were negatively correlated in SUS and NEXT_RS **(**Table 2).

The direction and strength of relationship between the toxicity, irritancy and repellency of MGI acaricides varied across the tested *T. urticae* strains. The toxicity and irritancy of clofentezine was significantly correlated in the SUS and NEXT_RS strains, while its toxicity and repellency were negatively correlated in the same strains but positively related in the APOLLO_RS strain. Irritancy and repellency of clofentezine was only positively related in the SUS strain **(**Table 3**)**.

**Table 3:** Correlation between contact toxicity, irritancy and repellency of mite growth inhibitor (MGI) acaricides on *T. urticae* strains.

Acaricide	*T. urticae strain*	Toxicity vs Irritancy		*P*	Toxicity vs Repellency		*P*	Irritancy Vs Repellency		*P*
		r (CI)	t*		r (CI)	t*		r (CI)	t*	
Clofentezine	SUS	**0.54 (−0.81** − **(−0.1))**	**−2.6**	**0.02**	**−0.74 (−0.90** − **(−0.41))**	**−4.4**	**0.0005**	**0.46 (−0.01–0.76)**	**2.1**	**0.05**
	Apollo_RS	−0.02 (−0.49–0.44)	−0.11	0.91	**0.46 (−0.01–0.76)**	**2.1**	**0.05**	−0.31 (−0.68–0.19)	−1.3	0.22
	Savey_RS	0.34 (−0.15–0.7)	1.5	0.16	0.43 (−0.04–0.75)	1.9	0.07	0.05 (−0.42–0.51)	0.21	0.83
	Zeal_RS	**0.47 (0.01–0.78)**	**2.1**	**0.05**	**−0.52 (0.79** − **(−0.07))**	**−2.4**	**0.03**	−0.31 (−0.68–0.19)	−1.3	0.22
	Next_RS	0.28 (−0.2–0.66)	1.2	0.25	0.28 (−0.22–0.66)	1.2	0.26	0.43 (−0.04–0.74)	1.9	0.07
Etoxazole	SUS	−0.26 (−0.64–0.23)	−1.1	0.29	0.18 (−0.71 − (−0.13))	−0.8	0.46	0.34 (−0.14–0.7)	1.5	0.16
	Apollo_RS	**0.46 (−0.01–0.76)**	2.1	**0.05**	−0.44 (−0.76–0.03)	−1.9	0.07	**−0.73 (−0.89** − **(−0.4))**	**−4.2**	**0.0006**
	Savey_RS	**−0.67 (−0.86** − **(−0.29))**	**−3.5**	**0.003**	−0.36 (−0.71–0.13)	−1.5	0.14	**0.51 (0.05–0.79)**	**2.4**	**0.03**
	Zeal_RS	−0.3 (−0.68–0.18)	−1.3	0.2	**−0.75 (−0.9** − **(−0.44))**	**−4.6**	**0.0003**	0.13 (−0.36–0.56)	0.5	0.61
	Next_RS	−0.34 (−0.64–0.26)	−1.5	0.16	0.13 (−0.36–0.56)	0.51	0.61	−0.18 (−0.59–0.31)	−0.74	0.47
Hexythiazox	SUS	−0.37 (−0.71–0.12)	−1.6	0.13	0.33 (−0.16–0.69)	1.4	0.18	**−0.58 (−0.13** − **(−0.71))**	**−2.8**	**0.01**
	Apollo_RS	−0.3 (−0.67–0.18)	−1.3	0.22	0.39 (−0.1–0.72)	1.7	0.11	0.18 (−0.31–0.59)	0.73	0.48
	Savey_RS	**0.75 (0.39–0.9)**	**4.1**	**0.001**	−0.30 (−0.68–0.19)	−1.3	0.22	−0.31 (−0.68–0.18)	−1.3	0.21
	Zeal_RS	**0.78 (0.5–0.92)**	**5.1**	**<0.0001**	**−0.58 (−0.83** − **(−0.14))**	**−2.7**	**0.01**	**−0.48 (−0.77** − **(−0.01))**	**−2.2**	**0.04**
	Next_RS	**0.5 (0.04–0.78)**	**2.3**	**0.03**	0.41 (−0.1–0.74)	1.8	0.09	0.36 (−0.13–0.7)	1.5	0.15

The toxicity and repellency of etoxazole were positively related in the APOLLO_RS and SAVEY_RS strains, while its toxicity and repellency were inversely related in Zeal-RS and APOLLO_RS. Irritancy and repellency of etoxazole were negatively related in SAVEY_RS **(**Table 3**)**.

The toxicity and irritancy of hexythiazox were positively related in SAVEY_RS, ZEAL_RS and Next_RS, while its toxicity and repellency were negatively related in ZEAL_RS. Irritancy and repellency were inversely related in SUS and NEXT_RS **(**Table 3**)**.

### Oviposition rate of *T. urticae* strains after contact with MET-I acaricides

Generally the oviposition rate of *T. urticae* strains was affected by dose of fenazaquin (*F*_*(2,120)*_ = 16.2, *P* < 0.0001), their resistance status (*F*_*(4,120)*_ = 7.3, *P* < 0.0001), strain*dose (*F*_*(8,120)*_ = 2.7, *P* = 0.01), and strain*dose*irritancy (*F*_*(8120)*_ = 2.6, *P* = 0.01), but not their irritancy response (*F*_*(1,120)*_ = 1.9, *P* = 0.17), dose*irritancy (*F*_*(2,120)*_ = 1.8, *P* = 0.17) and strain*irritancy (*F*_*(4,120)*_ = 0.66, *P* = 0.62). In the SUS strain, mites exposed to high doses of fenazaquin laid fewer eggs relative to other doses and irritated mites laid more eggs only at the low dose (*F*_*(5,24)*_ = 26.4, *P* = 0.0013). In the FUJI_RS strain irritated and non-irritated mites laid similar amount of eggs at low and high doses but irritated mites laid 1.5-fold more eggs compared to the non-irritated mites (*F*_*(5,24)*_ = 6.67, *P* = 0.0005). In NEXT_RS, irritated and non-irritated mites laid few eggs at high doses of fenazaquin relative to other doses and irritancy did affect the oviposition rate (*F*_*(5,24)*_ = 3.95, *P* = 0.0094). However, in MAG_RS there was no difference in the oviposition rate of irritated and non-irritated mites at middle and upper doses, but irritated mites laid 2-fold more eggs relative to non-irritated mites at the low dose of fenazaquin (*F*_*(5,24)*_ = 6.01, *P* < 0.001). In ZEAL_RS, at the low and middle doses of fenazaquin there was no differences between the oviposition rates of irritated and non-irritated mites, but at the high dose irritated mites laid more eggs than non-irritated mites (*F*_*(5,24)*_ = 7.2, *P* = 0.0003) (Supplementary Fig. 1).

Overall, the mites irritated by fenpyroximate laid more eggs relative to the non-irritated mites irrespective of their resistance status (strain) (*F*_(1, 118)_ = 51.97, *P* < 0.0001). The dose of fenpyroximate alone did not determine the oviposition rate of the mites (*F*_(1, 118)_ = 0.09, *P* = 0.92), but its interaction with irritancy (dose*irritancy *F*_(2, 118)_ = 4.96, *P* = 0.0086), strain (dose*strain *F*_(*8, 118*)_ = 3.5, *P* = 0.0086) and three way interaction with strain and irritancy (dose*strain*irritancy *F*_*(8, 118)*_ = 2.8, *P* = 0.006) were all significant. The reduced fecundity in non-irritated mites by fenpyroximate varied from one *T. urticae* strain to the other (strain*irritancy *F*_(8, 118)_ = 6.8, *P* < 0.0001). For example, in the SUS strain, fenpyroximate-irritated mites laid significantly more eggs at the median dose (4.4-fold) and the higher dose (20-fold) relative to non-irritated mites, but not at the low dose. While in the FUJI_RS, there was a 2.3 and 3.1-fold reduction in the oviposition rate at the median and upper dose respectively after continuous contact with fenpyroximate. Irritated mites consistently laid more eggs at all doses in the NEXT_RS strain. Irritated mites laid more eggs at the median (2.4-fold) and high (4.4-fold) dose of fenpyroximate in the MAG_RS strain. However in ZEAL_RS there was no significant difference in the oviposition rate between irritated and non-irritated mites at the tested doses of fenpyroximate (*F*_*(5,24)*_ = 1.9, *P* = 0.13) (Supplementary Fig. 2).

Across the tested *T. urticae* strains in this study, the resistance status (*F*_*(4,120)*_ = 32.7, *P* < 0.0001), irritancy (*F*_*(1,120)*_ = 15.5, *P* < 0.0001) and interactions between strain and dose (*F*_*(8,120)*_ = 8.9, *P* < 0.0001) had significant effects on the oviposition rate of *T. urticae* in contact with pyrabiden. In the SUS strain, irritated mites generally laid more eggs than the non-irritated mites at the moderate (1.5-fold) and high (3-fold) doses of pyrabiden. In the FUJI_RS, irritated mites laid 2-fold more eggs at the low and middle doses, and there was no irritancy effect on oviposition at the high dose. In NEXT_RS, there was no significant difference in the oviposition rate of irritated or non-irritated mites at high or low doses of pyrabiden, and irritated mites laid fewer (1.4-fold) eggs at the moderate dose. In MAG_RS, there was no difference in oviposition between irritated and non-irritated mites at the low and median doses, though irritated mites laid more (1.4-fold) eggs at high dose of pyrabiden. In ZEAL_RS, irritated and non-irritated mites had similar oviposition rates at the low and median doses of pyrabiden, but at the high dose irritated mites laid more eggs (4.9-fold) (Supplementary Fig. 3).

### Oviposition rate of *T. urticae* strains after contact with MGI acaricides

The resistant status of the tested *T. urticae* strains (*F*_*(4, 120)*_ = 9.7, *P* < 0.0001) and their irritancy (*F*_*(1, 120)*_ = 36.7, *P* < 0.0001) significantly affected mite oviposition rates. Interactions between the tested dose of clofentezine and *T. urticae* strain (*F*_*(8, 120)*_ = 17.8, *P* < 0.004) and between strain, dose, and irritancy (Strain*Dose* Irritancy, (*F*_*(8, 120)*_ = 1.9, *P* = 0.05) also affected the oviposition rates of the tested mites. Mites irritated by clofentezine tended to lay more eggs on average compared to unperturbed mites at the tested doses, especially in the SUS, APOLLO_RS and Next_RS strains (Supplementary Fig. 4).

The effect of hexythiazox on the fecundity of the tested *T. urticae* strains was significantly affected by the resistance status of the *T. urticae* strains (*F*_*(4, 120)*_ = 41, *P* < 0.0001), the dose of hexythiazox (*F*_*(2, 120)*_ = 5.4, *P* = 0.0005), and irritancy (*F*_*(1, 120)*_ = 78.1, *P* = 0.0001). Mite fecundity across the tested strains also depended on whether they were irritated or not by hexythiazox (strain*irritancy: _*(4, 24)*_ = 19.2, *P* = 0.0001), and the dose of hexythiazox applied (strain*irritancy*dose: *F*_*(8, 120)*_ = 4.3, *P* = 0.0001). Irritated mites laid more eggs compared to the non-irritated ones at similar dose of hexythiazox in the APOLLO_RS (excluding at high dose), SAVEY_RS, ZEAL_RS and Next_RS strains (Supplementary Fig. 5).

The oviposition rate of the tested *T. urticae* strains under treatment of etoxazole was significantly affected by the irritancy status (*F*_*(1, 120)*_ = 4.2, *P* = 0.04), the strain type (*F*_*(4, 120)*_ = 2.4, *P* = 0.05) and their interaction (strain*irritancy *F*_*(4, 120)*_ = 4, *P* = 0.005). The interaction between dose and strain also had a significant effect on the fecundity of the *T. urticae* strains (*F*_*(8, 120)*_ = 1.8, *P* = 0.09). In the SUS strain, irritated mites laid fewer eggs especially at high doses of etoxazole, while the reverse was mostly observed in the other *T. urticae* strains (Supplementary Fig. 6).

## Discussion

The outcome of this study demonstrates that non-neuro acting acaricides such as the MET-I acaricides (fenazaquin, fenpyroximate and pyrabiden) and MGIs (clofentezine, etoxazole and hexythiazox) can elicit significant behavioral responses via irritancy and repellency in *T. urticae* that can affect their contact toxicity. Furthermore, evolution of acaricide resistance can also modify the behavioral responses elicited in *T. urticae*. This has significant implications for the use of these acaricides in management of *T. urticae* populations in different cropping systems. Phytophagous mites such as *T. urticae* continue to pose an enormous threat to sustainable production of numerous crops. Hence, future evaluations of acaricide efficacy need to include behavioral responses of spidermites and not just acute toxicity of acaricides^16,20,28,29,37–43^. Since it is very possible to have incomplete spray coverage of acaricides in the field^5^, mites can easily disperse to untreated areas to cause population build ups and decrease the efficacy of acaricides.

The observed relative increase in fecundity of *T. urticae* strains after being irritated by acaricides highlights an ecological consequence that can easily lead to population increases or mite outbreaks in agroecosystems. This acaricide-induced oviposition has also been observed when *T. urticae* were exposed to doses of natural pesticides like colupulone^44^. Field studies have implicated this phenomenon in significant increases of mite populations^45,46^. It is thus possible that failure of applied acaricides to cause mite mortality after contact can result in mite outbreaks. A recent study^32^ observed that the abundance of *T. urticae* in commercial hop farms in Washington State was not correlated with their resistance status to fenpyroximate and etoxazole (mites were sampled after at least 1–2 field sprays). Thus, concerns about the spray coverage of acaricide application in cropping systems and pest control needs to be addressed.

In the absence of physiological resistance (i.e. SUS strain in this study), there is a direct link between toxicity and irritancy of the commercial formulations of fenazaquin, fenpyroximate and pyrabiden, and even for the MGI acaricide clofentezine. This suggests that *T. urticae* is neurologically sensitive to mortality-causing agents/compounds on contact. This can potentially affect the efficacy of these acaricides in the field, especially in the instance where acaricides are prophylactically sprayed and coverage is not uniform or effective. *T. urticae* can easily disperse to non-treated areas of the field or plant upon contact with acaricides. Interestingly, acaricide-susceptible mites have reduced ability to detect MET-I acaricides and clofentezine (negative correlation between toxicity and repellency). It is possible that phytophagous species like *T. urticae* are less effective at perceiving synthetic xenobiotics like acaricides, although there were cases where resistant *T. urticae* strains were repelled by acaricides even though they could overcome toxicity to exposure physiologically.

The similar mode of action of fenazaquin, fenpyroximate and pyrabiden was reflected in their low contact toxicity to the resistant strains FUJI_RS, MAG_RS and NEXT_RS, confirming previous reports of cross resistance among MET-I acaricides^47–50^. Similar cross resistance was observed among the MGI acaricides and their resistant strains. However, the behavioral response (irritancy and repellency) elicited by these seemingly similar acaricides varied significantly among the MET-I and MGI-resistant *T. urticae* strains. This clearly demonstrates that similar physiological effects of a group of acaricides does not translate to similar behavioral effects. Georghiou^26^ had postulated a negative correlation between physiological resistance and behavioral avoidance. However, this study found a mixed effect between the toxicity and behavioral response of *T. urticae* strains. For example, FUJI_RS is very resistant to MET-I acaricides but is still significantly irritated and repelled by MET-I acaricides. Based on the complexity of the observed relationship between toxicity, irritancy and repellency, it appears that physiological resistance and behavioral avoidance are not mutually synonymous and not mutually exclusive.

Formulated acaricides used commercially for mite control and also in our study usually contain inert ingredients that are meant to synergize the toxicity, solubility and stability of the active ingredients^51,52^. Currently, little is known on the effect of these non-active ingredients on the behavioral response of pest species. It is possible that the non-active ingredients in formulated acaricides also interact with *T. urticae*’s sensory system. Perhaps this explains the variation in the behavioral response of *T. urticae* strains of comparable resistance status to acaricides with similar mode of toxicity. Future studies should delineate between the behavioral effect of active and non-active acaricide ingredients^25^.

Though our study did not directly evaluate the chemosensory receptors and their mechanisms in *T. urticae*, its outcome, especially the variation in correlation between contact toxicity, repellency and irritancy across different strains of *T. urticae,* demonstrates that adaption to xenobiotics do alter the behavioral response in *T. urticae*. A similar phenomenon has been observed in pyrethroid-resistant aphid clones that exhibited a reduced response to their alarm pheromone^53^. The differential relationship between the behavioral responses to acaricides across the *T. urticae* strains strongly suggests complex assortment or segregation of chemosensory genes under acaricide-selection pressure in *T. urticae*. The *T. urticae* genome was recently discovered to harbor large numbers (689) of gustatory receptor genes^54^. However, how adaption to xenobiotics influences the expression of chemosensory genes is yet unknown. Future studies should investigate the relationship between chemosensory receptors and acaricide selection pressure.

Mechanisms of acaricide resistance mainly entail physiological changes such as target site mutations and enhanced metabolic detoxification that affects the toxico-dynamics and toxico-kinetics of the interaction between the active ingredients and target proteins^19^. The MGI and MET-I resistant *T. urticae* strains used in the study harbor the I1017F mutation in the CHS 1 gene^28^ and enhanced expression of cytochrome P450s (Adesanya *et al*. in prep) respectively, as the physiological mechanisms of acaricide resistance. Though the outcome of this study also suggests the presence of acaricide-avoidance behavior in these acaricide resistant strains, further study is required for temporal characterization of this behavioral attribute under acaricide selection pressure as outlined by Sparks *et al*.^24^ and Zalucki and Furlong^25^.

Accumulation of pesticide (acaricide) resistance alleles in arthropod species has been speculated to result in fitness change in the absence of selection pressure^55^. In most cases the fitness cost or advantage of acaricide resistance is usually evaluated by comparing life-history parameters in susceptible and resistant strains of the arthropod species in the absence of acaricide selection pressure. *T. urticae* strains with the I1017F and G314D and G326E mutations in the CHS 1 and glutamate–gated chloride channel were observed to have significant fitness costs^34^. Despite being physiologically resistant to acaricides, MET-I and MGI-resistant *T. urticae* strains in this study still exhibited acaricide-avoidance behavior via irritancy and repellency and also oviposited more on untreated areas after acaricide contact. This suggests that resistance to MET-I and MGI acaricides may have a negative effect beyond their target site. Future study should evaluate the impact of acaricide resistance development and treatment on the life history traits of *T. urticae* to provide further information for the management of acaricide resistance.

## Conclusion

The outcome of this study demonstrates that the acaricides fenazaquin, fenpyroximate, pyrabiden, clofentezine, hexythiazox, and etoxazole elicit behavioral responses in *T. urticae* that can affect their acute toxicity. The nature and degree of response elicited is highly dependent on the dose and physiology (resistance status) of *T. urticae*. This observation has a practical implication in the field management of *T. urticae*, especially in scenarios where there is ineffective spray coverage. The repellency/irritancy of a generalist herbivore can easily facilitate its dispersal to adjacent agroecosystems where it can cause further damage. Furthermore, the results of this study indicate the need for behavioral characterization of other acaricides, especially those with different modes of action in *T. urticae* and other phytophagous mite pests. Future studies are also needed to investigate the possible effects of acaricide formulation on behavioral response of *T. urticae* and other pest species, since the inert ingredients in acaricides most likely elicit sensory responses.

## Supplementary information

Supplementary Data

## Data Availability

The datasets generated and /or analyzed during this study are available from the corresponding author on reasonable request.
