# Supplementary material for: Physiological resistance alters behavioral response of Tetranychus urticae to acaricides
Source: Sci Rep. 2019 Dec 17;9:19308. doi: 10.1038/s41598-019-55708-4 (PMC6917710; doi:10.1038/s41598-019-55708-4)
Supplement: Supplementary file 1 — Supplementary Data [file 41598_2019_55708_MOESM1_ESM.pdf]

Physiological resistance alters behavioral response of *Tetranychus urticae* to acaricides

Adekunle W. Adesanya[1,2], Michael J. Beauchamp[1], Mark D. Lavine[2], Laura C. Lavine[2], Fang Zhu[2,3] and Doug B.Walsh[1,2]

Supplementary Data

[1] Irrigated Agriculture Research and Extension Center, Washington State University, Prosser, WA 99350, USA

[2] Department of Entomology, Washington State University, Pullman, WA 99164, USA.

[3] Department of Entomology, Pennsylvania State University, State College, PA 16803

Supplementary Figure 1

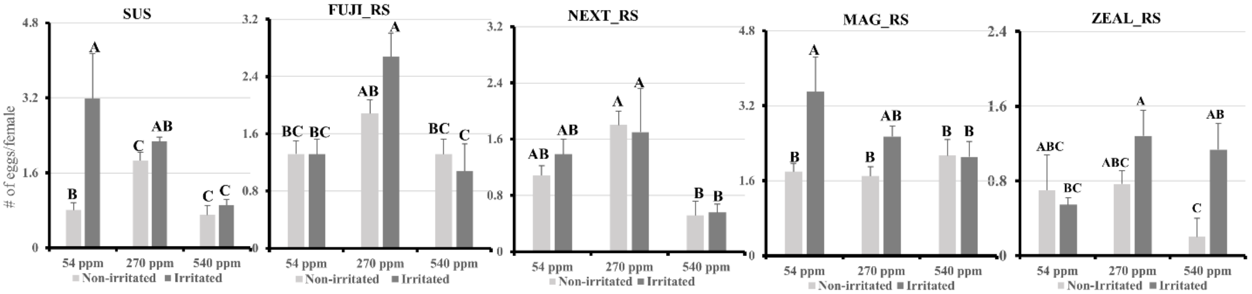

SUS: $F_{(5,24)}$=5.7, P=0.001

Source	DF	F Ratio	P > F
Dose	2	5.8	0.01
Irritancy	1	16.4	0.001
Dose*Irritancy	2	4.2	0.02

FUJI_RS: $F_{(5,24)}$=5.63, P=0.08

Source	DF	F Ratio	P > F
Dose	2	11.9	0.0003
irritancy	1	0	1
Dose*irritancy	2	1.01	0.4

NEXT_RS: $F_{(5,24)}$=3.4, P=0.019

Source	DF	F Ratio	P > F
Dose	2	8.1	0.002
irritancy	1	0.5	0.01
Dose*irritancy	2	0.2	0.8

MAG_RS: $F_{(5,24)}$=2.95, P=0.03

Source	DF	F Ratio	P > F
Dose	2	1.2	0.3
irritancy	1	9.8	0.01
Dose*irritancy	2	2.5	0.1

ZEAL_RS: $F_{(5,24)}$=2.5, P=0.052

Source	DF	F Ratio	P > F
Dose	2	1.6	0.2
irritancy	1	0.2	0.7
Dose*irritancy	2	2.5	0.1

Supplementary figure 1: The effect of **fenazaquin** and its irritancy on the oviposition rate of *T. urticae* strains

Supplementary Figure 2

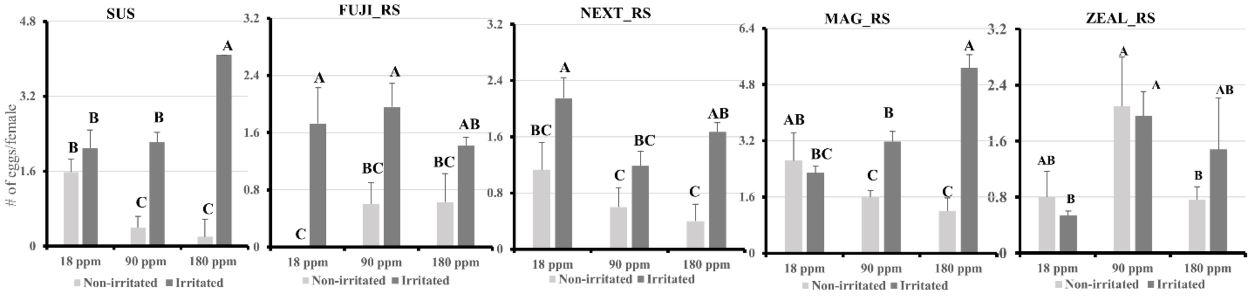

$F_{(5,24)}$ =26.4 P<0.0001 $F_{(5,24)}$=4.1, P=0.08 $F_{(5,24)}$ =6.1, P=0.001 $F_{(5,24)}$ =2.6, P=0.05 $F_{(5,24)}$ =1.9, P=0.1

Source	DF	F Ratio	P > F
Dose	2	3.5	0.04
Irritancy	1	1.6	0.2
Dose*Irritancy	2	20.2	<0.0001

Source	DF	F Ratio	P > F
Dose	2	0.6	0.5
irritancy	1	10.6	0.003
Dose*irritancy	2	0.8062	0.5

Source	DF	F Ratio	P > F
Dose	2	4.9	0.02
irritancy	1	7.3	0.01
Dose*irritancy	2	1.2	0.3

Source	DF	F Ratio	P > F
Dose	2	1.8	0.2
irritancy	1	0.9	0.3
Dose*irritancy	2	4.1	0.03

Source	DF	F Ratio	P> F
Dose	2	4.1	0.03
irritancy	1	0.1	0.7
Dose*irritancy	2	0.6	0.6

Supplementary figure 2: The effect of **fenpyroximate** and its irritancy on the oviposition rate of *T. urticae* strains

Supplementary Figure 3

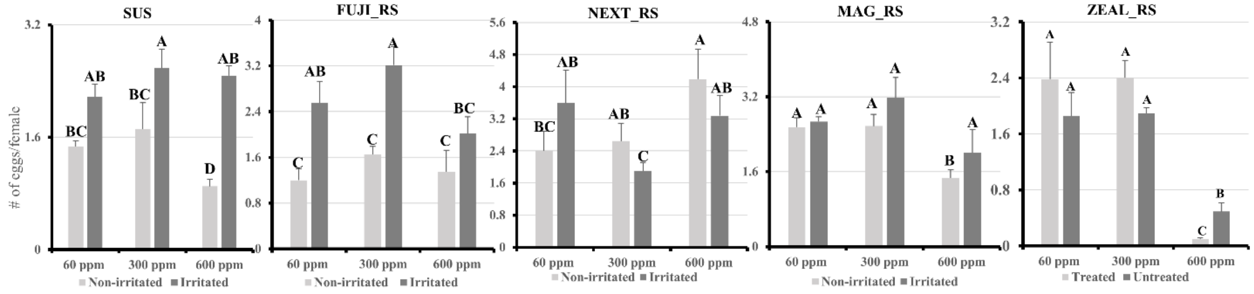

$F_{(5,24)}$ =3.9, P=0.007 $F_{(5,24)}$=6.67, P=0.0005 $F_{(5,24)}$ =3.95, P=0.009 $F_{(5,24)}$ =6.01, P<0.001 $F_{(5,24)}$ =7.2, P=0.0003

Source	DF	F ratio	P > F
Dose	2	0.8	0.46
Irritancy	1	3.8	0.0005
Dose*Irritancy	2	0.4	0.7

Source	DF	F ratio	P > F
Dose	2	3.4	0.05
irritancy	1	10.3	0.004
Dose*irritancy	2	1.2	0.3

Source	DF	F ratio	P > F
Dose	2	1.7	0.2
irritancy	1	7.9	0.002
Dose*irritancy	2	1.5	0.2

Source	DF	F ratio	P> F
Dose	2	2.2	0.1
irritancy	1	0.1	0.7
Dose*irritancy	2	5.205	0.01

Source	DF	F ratio	P > F
Dose	2	15.9	< 0.0001
irritancy	1	2.9	0.1
Dose*irritancy	2	1.2	0.3

Supplementary figure 3: The effect of **pyrabiden** and its irritancy on the oviposition rate of *T. urticae* strains

Supplementary Figure 4

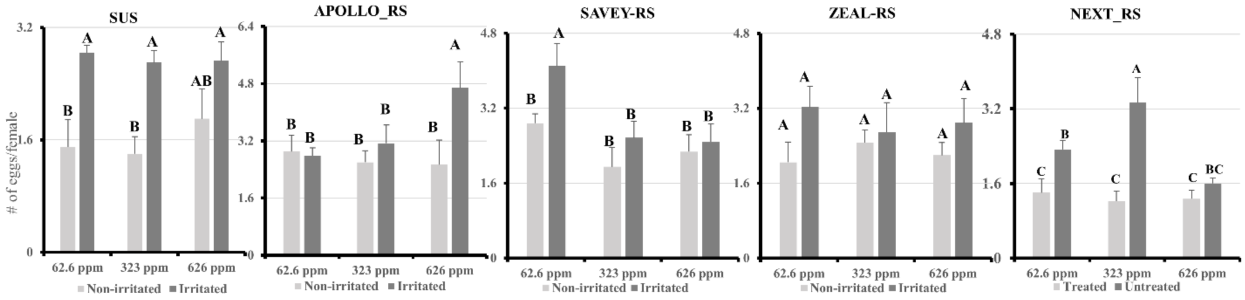

$F_{(5,24)}$ =6.4, P=0.0006 $F_{(5,24)}$=2.2, P=0.08 $F_{(5,24)}$ =3.9, P=0.01 $F_{(5,24)}$ =1.01, P=0.4 $F_{(5,24)}$ =7.9, P=0.0002

Source	DF	F ratio	P > F
Dose	2	1.9	0.18
Irritancy	1	25.1	<0.0001
Dose*Irritancy	2	3.2	0.08

Source	DF	F ratio	Pr > F
Dose	2	1.8	0.2
irritancy	1	3.5	0.07
Dose*irritancy	2	2.04	0.15

Source	DF	F ratio	P > F
Dose	2	6.4	0.006
irritancy	1	6.4	0.04
Dose*irritancy	2	0.8	0.4

Source	DF	F ratio	P> F
Dose	2	0.4	0.7
irritancy	1	3.1	0.1
Dose*irritancy	2	0.6	0.54

Source	DF	F ratio	P > F
Dose	2	1.9	0.18
irritancy	1	25.1	<0.0001
Dose*irritancy	2	3.2	0.08

Supplementary figure 4: The effect of **clofentezine** and its irritancy on the oviposition rate of *T. urticae* strains

Supplementary Figure 5

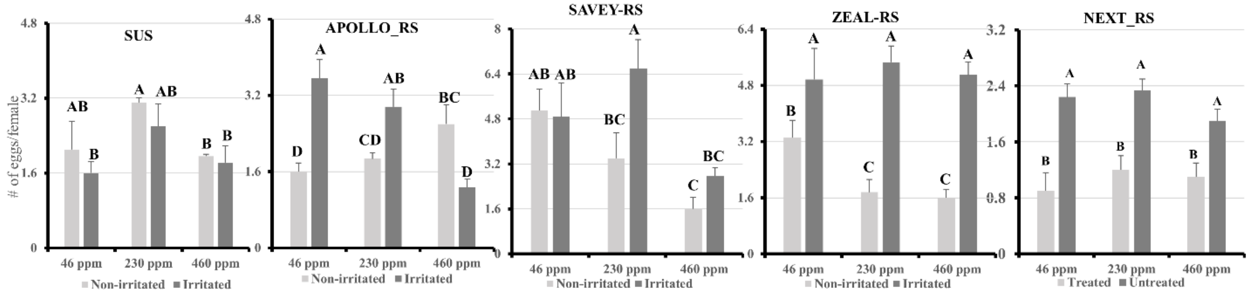

$F_{(5,24)}$ =2.4 P=0.07 $F_{(5,24)}$=8.5, P<0.0001 $F_{(5,24)}$ =4.4, P=0.006 $F_{(5,24)}$=11.3, P<0.0001 $F_{(5,24)}$ =8.8, P<0.0001

Source	DF	F ratio	P > F
Dose	2	5.0	0.01
Irritancy	1	1.4	0.25
Dose*Irritancy	2	0.17	0.87

Source	DF	F ratio	P> F
Dose	2	2.5	0.1
irritancy	1	5.5	0.03
Dose*irritancy	2	16.1	<0.0001

Source	DF	F ratio	P > F
Dose	2	7.2	0.004
irritancy	1	3.8	0.06
Dose*irritancy	2	1.9	0.17

Source	DF	F ratio	P> F
Dose	2	1.3	0.3
irritancy	1	39.3	<0.0001
Dose*irritancy	2	2.4	0.1

Source	DF	F ratio	P > F
Dose	2	1.2	0.3
irritancy	1	39.3	<0.0001
Dose*irritancy	2	1.1	0.3

Supplementary figure 5: The effect of **hexythiazox** and its irritancy on the oviposition rate of *T. urticae* strains

Supplementary Figure 6

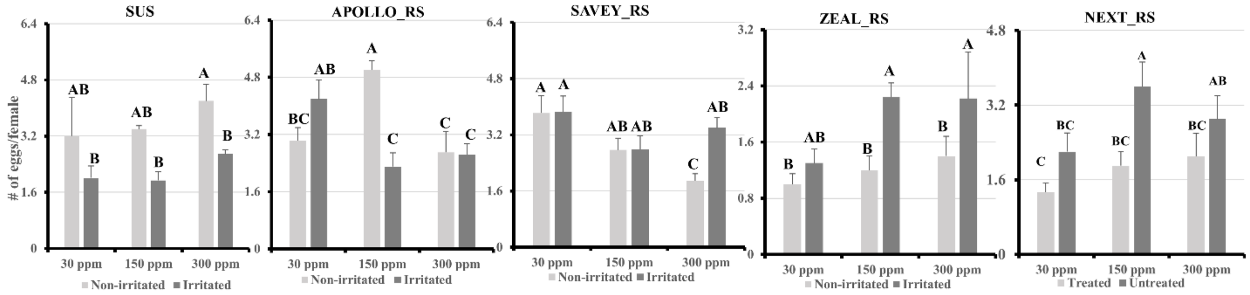

SUS: $F_{(5,24)}=3.1$, $P=0.03$

Source	DF	F ratio	P > F
Dose	2	1.8	0.2
Irritancy	1	11.4	0.003
Dose*Irritancy	2	0.08	0.9

APOLLO_RS: $F_{(5,24)}=5.63$, $P=0.001$

Source	DF	F ratio	P > F
Dose	2	3.4	0.05
irritancy	1	10.3	0.004
Dose*irritancy	2	1.23	0.3

SAVEY_RS: $F_{(5,24)}=3.9$, $P=0.01$

Source	DF	F ratio	P > F
Dose	2	5.9	0.01
irritancy	1	2.6	0.1
Dose*irritancy	2	2.7	0.1

ZEAL_RS: $F_{(5,24)}=2.5$, $P=0.05$

Source	DF	F ratio	P> F
Dose	2	2.1	0.2
irritancy	1	7.3	0.01
Dose*irritancy	2	0.53	0.6

NEXT_RS: $F_{(5,24)}=3.5$, $P=0.02$

Source	DF	F ratio	P > F
Dose	2	3.01	0.07
irritancy	1	10.6	0.003
Dose*irritancy	2	0.6	0.6

Supplementary figure 6: The effect of **etoxazole** and its irritancy on the oviposition rate of *T. urticae* strains

Supplementary table 1: number of mites from each strain tested for toxicity(mortality)

Acaricide Class	Acaricide	Strain	Low dose	Moderate Dose	High Dose
MET-I	Fenazaquin	SUS	90	85	75
		FUJI_RS	73	69	72
		NEXT_RS	66	62	71
		MAG_RS	66	71	70
		ZEAL_RS	70	81	70
MET-I	Fenpyroximate	SUS	85	84	78
		FUJI_RS	69	66	73
		NEXT_RS	80	64	73
		MAG_RS	68	82	73
		ZEAL_RS	65	67	67
MET-I	Pyrabiden	SUS	77	80	83
		FUJI_RS	81	63	77
		NEXT_RS	70	71	87
		MAG_RS	69	75	76
		ZEAL_RS	71	71	81
MGI	Clofentezine	SUS	197	228	236
		APOLLO_RS	196	167	131
		SAVEY_RS	137	102	100
		ZEAL_RS	111	111	118
		NEXT_RS	165	138	174
MGI	Hexythiazox	SUS	113	190	122
		APOLLO_RS	140	142	142
		SAVEY_RS	119	123	152
		ZEAL_RS	188	122	153
		NEXT_RS	152	202	114
MGI	Etoxazole	SUS	175	203	163
		APOLLO_RS	210	195	155
		SAVEY_RS	167	161	259
		ZEAL_RS	230	261	188
		NEXT_RS	100	190	171

Supplementary table 2: number of mites from each strain tested for irritancy

Acaricide Class	Acaricide	Strain	Low dose	Moderate Dose	High Dose
MET-I	Fenazaquin	SUS	47	47	56
		FUJI_RS	48	43	41
		NEXT_RS	43	23	46
		MAG_RS	49	45	48
		ZEAL_RS	39	45	43
MET-I	Fenpyroximate	SUS	41	48	45
		FUJI_RS	32	34	32
		NEXT_RS	35	31	35
		MAG_RS	44	0.32	48
		ZEAL_RS	29	38	36
MET-I	Pyrabiden	SUS	39	52	55
		FUJI_RS	36	51	38
		NEXT_RS	55	72	50
		MAG_RS	44	43	38
		ZEAL_RS	44	38	32
MGI	Clofentezine	SUS	42	43	38
		APOLLO_RS	44	39	31
		SAVEY_RS	42	37	37
		ZEAL_RS	40	40	39
		NEXT_RS	42	43	40
MGI	Hexythiazox	SUS	44	45	53
		APOLLO_RS	37	41	40
		SAVEY_RS	45	45	53
		ZEAL_RS	48	52	47
		NEXT_RS	50	53	51
MGI	Etoxazole	SUS	55	72	53
		APOLLO_RS	44	42	48
		SAVEY_RS	39	44	44
		ZEAL_RS	43	47	53
		NEXT_RS	44	50	50

Supplementary table 3: number of mites from each strain tested for repellency

Acaricide Class	Acaricide	Strain	Low dose	Moderate Dose	High Dose
MET-I	Fenazaquin	SUS	41	40	35
		FUJI_RS	25	37	47
		NEXT_RS	41	42	34
		MAG_RS	35	37	42
		ZEAL_RS	23	49	37
MET-I	Fenpyroximate	SUS	48	48	51
		FUJI_RS	48	42	44
		NEXT_RS	33	40	41
		MAG_RS	36	40	37
		ZEAL_RS	45	41	41
MET-I	Pyrabiden	SUS	52	57	51
		FUJI_RS	38	40	40
		NEXT_RS	44	44	40
		MAG_RS	39	39	40
		ZEAL_RS	41	47	43
MGI	Clofentezine	SUS	60	57	49
		APOLLO_RS	43	40	51
		SAVEY_RS	40	46	51
		ZEAL_RS	41	41	42
		NEXT_RS	39	41	41
MGI	Etoxazole	SUS	45	46	32
		APOLLO_RS	45	41	44
		SAVEY_RS	42	44	41
		ZEAL_RS	34	40	32
		NEXT_RS	50	55	52
MGI	Hexythiazox	SUS	29	37	34
		APOLLO_RS	32	34	41
		SAVEY_RS	47	37	36
		ZEAL_RS	47	39	45
		NEXT_RS	41	31	36
